# Revealing the impact of psychiatric comorbidities on treatment outcome in early psychosis using counterfactual model explanation

**DOI:** 10.3389/fpsyt.2023.1237490

**Published:** 2023-10-12

**Authors:** Violet van Dee, Seyed Mostafa Kia, Inge Winter-van Rossum, René S. Kahn, Wiepke Cahn, Hugo G. Schnack

**Affiliations:** ^1^Department of Psychiatry, Brain Center, University Medical Center Utrecht, Utrecht University, Utrecht, Netherlands; ^2^Donders Institute for Brain, Cognition and Behaviour, Radboud University, Nijmegen, Netherlands; ^3^Department of Cognitive Science and Artificial Intelligence, Tilburg University, Tilburg, Netherlands; ^4^Icahn School of Medicine at Mount Sinai, New York City, NY, United States; ^5^Altrecht Science, Altrecht Mental Health Institute, Utrecht, Netherlands

**Keywords:** psychosis, comorbidity, precision psychiatry, machine learning, counterfactual explanation

## Abstract

**Introduction:**

Psychiatric comorbidities have a significant impact on the course of illness in patients with schizophrenia spectrum disorders. To accurately predict outcomes for individual patients using computerized prognostic models, it is essential to consider these comorbidities and their influence.

**Methods:**

In our study, we utilized a multi-modal deep learning architecture to forecast symptomatic remission, focusing on a multicenter sample of patients with first-episode psychosis from the OPTiMiSE study. Additionally, we introduced a counterfactual model explanation technique to examine how scores on the Mini International Neuropsychiatric Interview (MINI) affected the likelihood of remission, both at the group level and for individual patients.

**Results:**

Our findings at the group level revealed that most comorbidities had a negative association with remission. Among them, current and recurrent depressive disorders consistently exerted the greatest negative impact on the probability of remission across patients. However, we made an interesting observation: current suicidality within the past month and substance abuse within the past 12 months were associated with an increased chance of remission in patients. We found a high degree of variability among patients at the individual level. Through hierarchical clustering analysis, we identified two subgroups: one in which comorbidities had a relatively limited effect on remission (approximately 45% of patients), and another in which comorbidities more strongly influenced remission. By incorporating comorbidities into individualized prognostic prediction models, we determined which specific comorbidities had the greatest impact on remission at both the group level and for individual patients.

**Discussion:**

These results highlight the importance of identifying and including relevant comorbidities in prediction models, providing valuable insights for improving the treatment and prognosis of patients with psychotic disorders. Furthermore, they open avenues for further research into the efficacy of treating these comorbidities to enhance overall patient outcomes.

## 1. Introduction

Psychiatric comorbidity is highly prevalent in schizophrenia spectrum disorders (SSD). Among a cohort of 1,446 schizophrenia patients, 56% had at least one additional mental health disorder (1). Substance use disorders were found in 42% of patients (2), anxiety disorders in 38% (3), major depressive disorders in 32% (4), ADHD in 10%–60% (5), and autism spectrum disorders in 3.6% (6). Previous studies primarily focused on examining individual diagnoses in SSD, despite the complexity of comorbidities extending beyond dualities. This may be attributed to genetic correlations between psychiatric disorders (7), symptom overlap within classification systems, and the significant impact of having SSD and receiving treatment for it (iatrogenic comorbidity) on overall mental health.

Psychiatric comorbidity in SSD serves as a negative prognostic factor. Prior research has demonstrated that the presence of comorbid mental health disorders in patients with SSD is associated with reduced symptomatic improvement (3, 8), higher readmission rates (4, 9), poorer functioning (3), lower quality of life (10), and increased suicidality (11). Specifically, comorbid anxiety disorders have been linked to worse initial outcomes in first-episode psychosis, more severe clinical features in later stages of schizophrenia, and poorer functioning (3). Obsessive compulsive disorder has been associated with more positive and global psychotic symptoms in patients with schizophrenia (12). Post-traumatic stress disorders have been associated with higher levels of positive and general symptoms, more neurocognitive impairment, worse functioning and lower quality of life (13). Comorbid depressive symptoms and depressive disorders are associated with a higher number of lifetime hospitalizations (4), reduced chances of functional remission (14), lower quality of life (10, 15), and increased suicidality (11). Moreover, comorbid substance use disorders are linked to more positive symptoms (16), more frequent clinical exacerbations, impaired global functioning and higher relapse rates (17), increased readmissions (4, 9), and elevated suicide attempts (11).

Machine learning (ML) has emerged as a valuable tool for outcome prediction in early psychosis (18). By analyzing extensive clinical data, ML algorithms can identify intricate patterns that may elude human perception, including subtle interactions among symptoms, demographics, and other factors. In clinical applications, it is essential to explain the decisions made by complex ML models. The model explanation provides insights into the most influential features or variables guiding predictions, enhancing transparency and comprehensibility for non-experts, and facilitating communication between clinicians and patients.

One promising approach for interpreting complex ML models is the counterfactual model explanation (19). This technique involves generating hypothetical instances that would result in different outcomes, shedding light on the model’s decision-making process. What sets counterfactual interpretation apart is its ability to explain individual patient-level decisions, making it suitable for precision medicine applications. By leveraging the power of ML and employing a counterfactual model explanation, we aim to assess the role of psychiatric comorbidity as a predictor of SSD outcomes. This research contributes to the advancement of personalized prognostic prediction models by introducing a new ML pipeline that enables more accurate and tailored treatment for patients with SSD.

## 2. Materials and methods

### 2.1. OPTiMiSE dataset

In this study, we use the OPTiMiSE dataset (20) that has been collected in a multicenter antipsychotic three-phase switching study. In this dataset, the patients with first-episode psychosis were examined at multiple visits and across three phases of the study in which the patients were treated with three antipsychotic medications: amisulpride, olanzapine, and clozapine. In our experiments, we used the data from the first two phases of the study. 446 patients were enrolled in this study. In the first phase, the patients were treated with amisulpride for four weeks. At the end of the first phase, the patients who met Andreasen’s symptomatic remission criterion (21) were excluded. 371 patients completed the first phase. The rest of the patients went on to the second phase and, after randomization, either continued using amisulpride or switched to olanzapine for another six weeks. Among 72 patients who completed the second phase, 66 patients without missing PANSS records were used in our analysis.

Of these 66 patients, 79% are males with a mean (SD) age of 25.3 (0.8) years. Most of them had a DSM classification of schizophrenia (67%) or schizophreniform disorders (32%) and one patient had a schizoaffective disorder (2%). At baseline, 55% of the patients were admitted while 45% were outpatients. The mean (SD) duration of the current psychotic episode was 2.5 (0.5) months. Only 32% of the patients had (volunteer)work or school at baseline. 30% of the patients suffered from one or more psychiatric comorbidities in the present or past; 21% of them had one or more mood disorders, 18% had one or more anxiety disorders, and 5% had one or more substance use disorders. In addition, 20% had been suicidal in the past month.

We used a set of 179 measures (see Table 1) as predictors in our psychosis prognosis prediction model. This set of inputs is a mixture of continuous, categorical, and binary variables with missing values. For continuous variables, we used the median to impute the missing values. Then a robust min-max scaler is employed to rescale the measures to the range of (0,1). The missing binary and categorical variables are imputed with the most frequent category. The categorical variables are then one-hot encoded. For binary variables (such as the presence or absence of comorbidity by the MINI), a simple −1/1 encoding is used.

**Table 1:** The type, number, and list of features from the OPTiMiSE study that are used as predictors in our model.

Type	Number of features	Features	Visits
Demographic	20	Age (con), Sex (bin), Race (cat), Immigration status (bin), Marital status (bin), Divorce status (bin), Occupation status (bin), Occupation type (cat), Previous occupation status (bin), Previous occupation type (cat), Father’s occupation (cat), Mother’s occupation (cat), Years of education (con), Highest education level (cat), Father’s highest degree (cat), Mother’s highest degree (cat), Living status (bin), Dwelling (cat), Income source (cat), Living environment (cat)	Baseline
Diagnostic	7	DSM-IV classification (cat), Duration of the current psychotic episode (con), Current psychiatric treatment (cat), Psychosocial interventions status (bin), Estimated prognosis (cat), Hospitalization status (bin)	Baseline
Lifestyle	7	Recreational drugs history (bin), Recreational drugs since last visit (bin), Caffeine drinks per day (con), Last caffeine drink (cat), Drink Alcohol (bin), Alcoholic drinks in the last year (cat), Smoking status (bin)	Baseline
Somatic	11	Height (con), Weight (con), Waist (con), Hip (con), BMI (con), Systolic blood pressure (con), Distolic blood pressure (con), Pulse (con), ECG abnormality (bin), Last mealtime (cat), Last meal type (cat)	Baseline
Treatment	1	Average medication dosage (con)	Baseline
CDSS	9	Calgary Depression Scale for Schizophrenia (con)	Baseline
SWN	20	Subjective Well-being under Neuroleptic Treatment Scale (con)	Baseline
MINI	48	Mini International Neuropsychiatric Interview	Baseline
PANSS	30	Positive And Negative Symdrome Scale (con)	Baseline, Weeks 2, 3, 5, 6, 8
PSP	5	Personal and Social Performance Scale (con)	Baseline, Weeks 2, 3, 5, 6, 8
CGI	2	Clinical Global Impression Scale severity and improvement (con)	Baseline, Weeks 2, 3, 5, 6, 8

Con, continuous measure; bin, binary measure; cat, categorical measure.

### 2.2. Analysis pipeline

**Figure 1:**
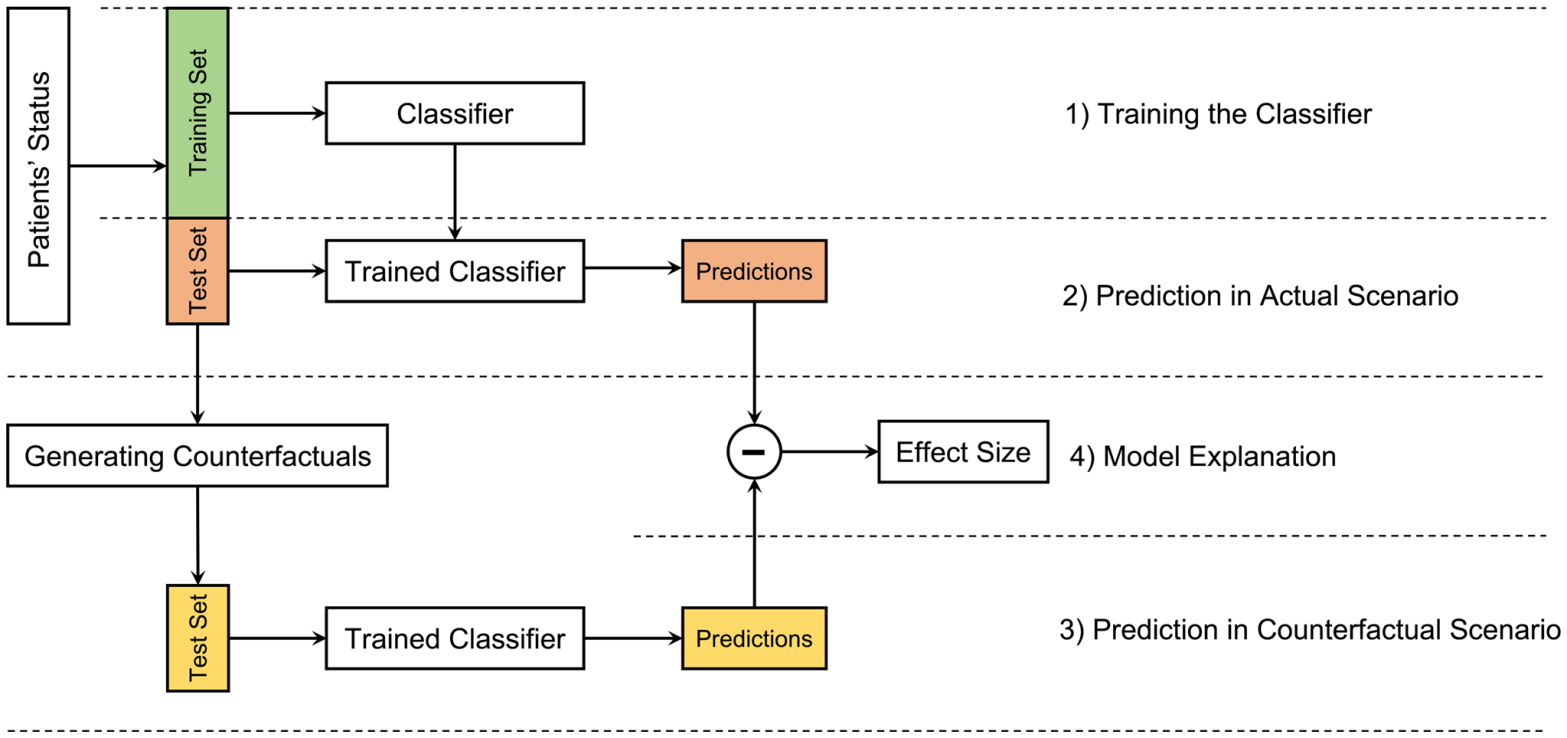
An overview of the analysis pipeline. The pipeline includes four steps: (1) after splitting the patients’ status into the training and the test set (for example, using K-fold cross-validation) a classifier is trained on the training set to predict the symptomatic remission of patients; (2) the trained classifier is used to predict symptomatic remission of patients in the test set in an actual scenario, i.e., on real data; (3) after generating counterfactual versions of samples in the test set, the predictions in the counterfactual scenarios are computed; and (4) by computing the difference between the predicted probabilities for symptomatic remission in the actual and counterfactual scenarios the effect of each comorbidity feature on the final prediction is evaluated for a further model explanation.

Figure 1 depicts an overview of the analysis pipeline introduced in this study. The pipeline includes four steps: (1) training the classifier, (2) prediction of the probability of symptomatic remission in the actual scenario in which the patient’s comorbidity status is kept unchanged, (3) prediction of the probability of symptomatic remission in the counterfactual scenario in which the patient’s comorbidity status is changed by flipping a comorbidity feature, and (4) model explanation by computing the effect of each comorbidity measure on the final predicted probabilities. The subsequent sections will delve into a detailed description of each step, elucidating the methodologies and processes involved.

#### 2.2.1. Step 1: training the classifier and making predictions in actual scenarios

**Figure 2:**
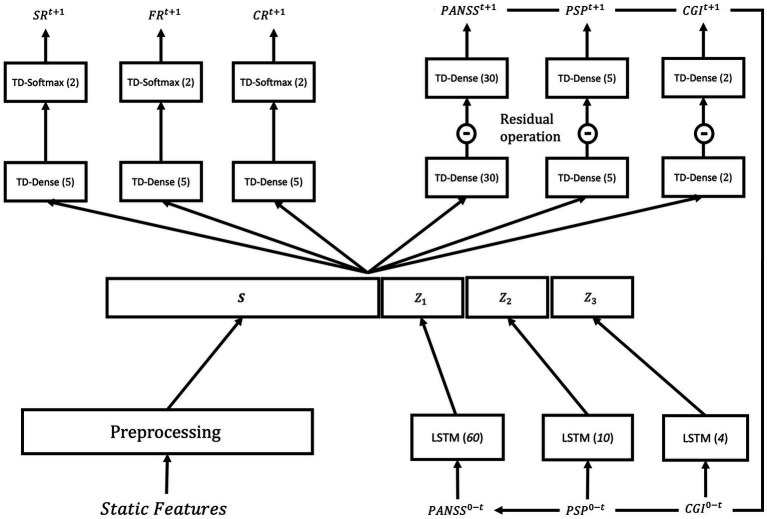
The neural network architecture from van Opstal et al. (22) that is adopted in this study. The architecture consists of (1) modality-specific LSTM layers that learn a middle representation for dynamic (time-varying) features including PANSS, PSP, and CGI from timepoint 0 to *t*; (2) a fusion layer that merges the preprocessed static features (which do not change over time) with dynamic middle representations; (3) the interaction layer consisting of outcome-specific time-distributed dense layers that seek to benefit from interaction between static features and dynamic features from different modalities; and (4) the output layer with time-distributed softmax and dense layers that predict the outputs at the next time step *t + 1* (SR, symptomatic remission; FR, functional remission; CR, clinical remission).

In this study, we adopted a recurrent deep neural network architecture proposed by van Opstal et al. (22) for predicting symptomatic remission. The architecture is depicted in Figure 2. This architecture effectively handles both dynamic and static patient statuses, making it well-suited for psychosis prognosis prediction using the OPTiMiSE dataset, which includes a combination of dynamic (e.g., PANSS, PSP, CGI) and static features (e.g., demographic and comorbidity features).

The architecture begins by transforming the dynamic features into a latent representation using long short-term memory (LSTM) units. This latent representation is then merged with the static features and fed into a series of fully connected layers to produce the final prediction. A comprehensive description of the model architecture and training procedure can be found in van Opstal et al. (22), and we followed the same training procedure outlined in that work. The training procedure encompasses feature preprocessing, pretraining on synthesized data, data augmentation, training on augmented data, and model calibration. During the training process, a multi-objective loss function is optimized, incorporating weighted mean squared and categorical cross-entropy loss functions for regression and classification tasks, respectively. To facilitate the optimization, an Adam optimizer with an initial learning rate of $3\times 10^{-4}$ is utilized. Additionally, an exponential learning rate decay with a decay rate of 0.9 and decay steps of 10,000 is applied. In the pretraining phase, the network undergoes training for two epochs, employing a mini-batch size of 25 samples. Subsequently, during the training phase, the network is further trained on augmented data for 50 epochs, using a mini-batch size of 2 samples [see van Opstal et al. (22) for more details about the architecture and training procedures].

To evaluate the prediction model’s performance, we conducted 20 repetitions of 10-fold and one-site-out cross-validation. The classification model was assessed using various metrics, including the area under the receiver operating characteristic curve (AUC), balanced accuracy (BAC), sensitivity, and specificity. To assess the impact of including the MINI-PLUS features on the model’s performance in predicting symptomatic remission, we trained the model both with and without the MINI-PLUS items as inputs.

#### 2.2.2. Steps 2–4: prediction in counterfactual scenarios and model explanation

Here, we introduce a counterfactual model explanation technique (19) to study the effect of comorbidity on the chance of symptomatic remission of patients. The counterfactual model explanation is an emerging technique for explaining decisions of complex “black-box” models. A counterfactual explanation defines a causal scenario by envisioning an alternative reality $X^{\prime }$ for a specific event X , leading to a different outcome. This type of reasoning allows us to understand the causal relationships between events and outcomes. In machine learning, counterfactual explanations are used to clarify *individual* predictions. By generating counterfactual examples, where feature values are changed, we can analyze how predictions respond, providing valuable insights for various applications, such as treatment outcome prediction (23).

For example, consider a medical scenario where a machine learning model predicts treatment outcomes for patients. The event is the predicted outcome, like successful recovery or adverse effects, and the causes are specific patient features, such as age, medical history, and treatment protocol. Generating counterfactual explanations involves altering the feature values of a patient to explore different treatment scenarios and analyze how the prediction changes. This approach can help medical practitioners understand the critical factors that influence treatment success and identify potential improvements for better patient outcomes.

**Figure 3:**
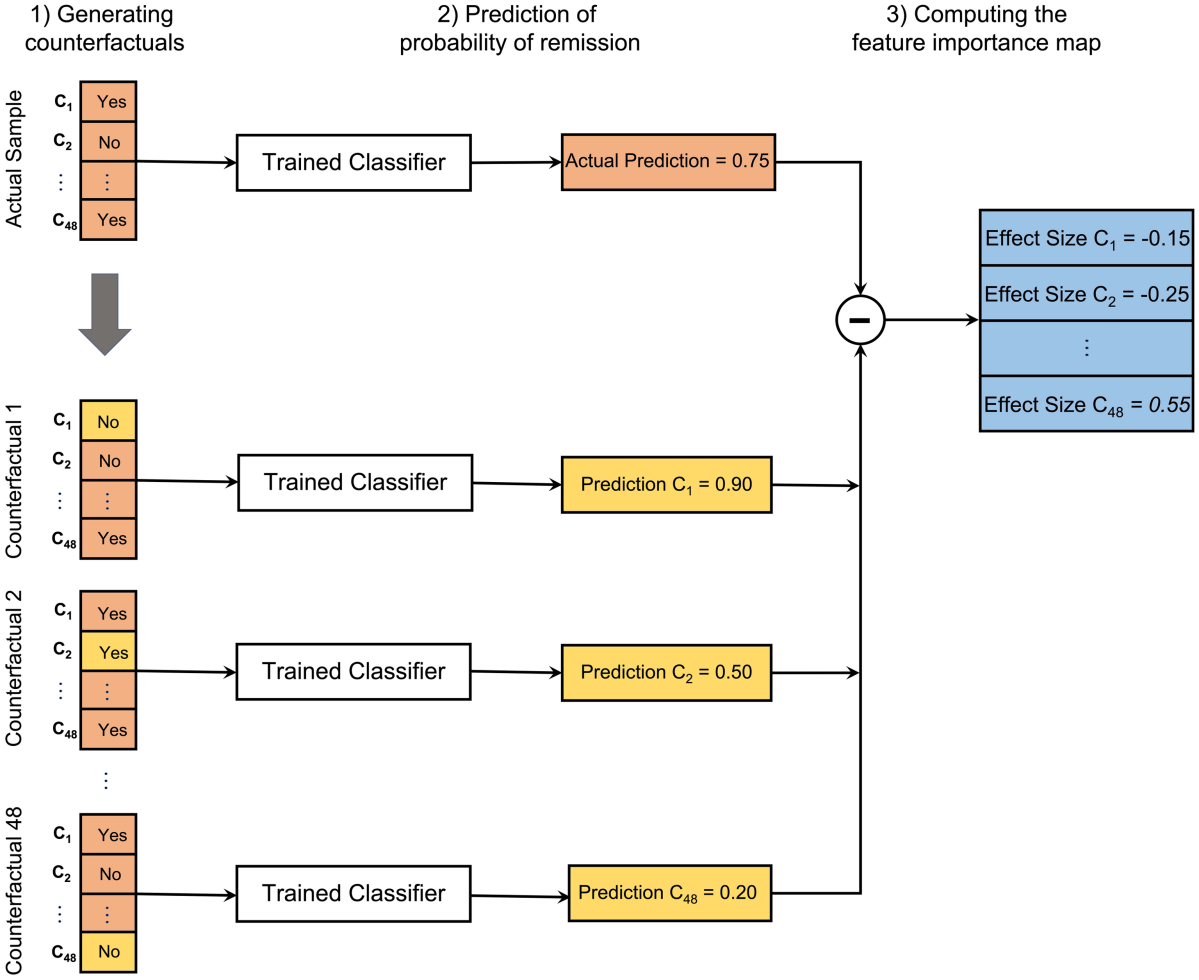
A schematic of the model explanation procedure in three steps: (1) For an actual sample (in the test set) with 48 binary MINI items (*C_1_, C_2_,…, C_48_*), 48 counterfactuals are generated where in each counterfactual sample only one binary feature value is flipped from “yes” to “no” or from “no” to “yes” (colored in yellow). (2) The probabilities of symptomatic remission for the actual and 48 counterfactual samples are predicted by the trained classifier. (3) The feature importance map for a given patient is compiled by computing the effect size for every 48 comorbidity features. The effect size for feature *C_i_* is computed by subtracting the predicted probability of remission in the actual/counterfactual scenario in which *C_i_ = “no”* from the predicted probability of remission in the counterfactual/actual scenario in which *C_i_ = “yes”*.

In this study, we introduce a counterfactual model technique to evaluate the effect of psychiatric comorbidity from the Mini International Neuropsychiatric Interview PLUS (MINI-PLUS) on symptomatic remission in first-episode psychosis. To this end, we used MINI-PLUS without the schizophrenia spectrum disorders items, resulting in 48 binary entries (see Supplementary Table S1 for the complete list). The effect of each MINI item on the probability of symptomatic remission of an individual patient, i.e., the feature importance map, is computed in three steps (see Figure 3):

1. Generating the counterfactuals: given the actual 48 binary MINI items (*C_1_, C_2_,…, C_48_*) for a specific patient in the test set, 48 counterfactual samples are generated. For each counterfactual sample, exactly one binary item is flipped from “yes” to “no” or “no” to “yes”. The rest of the items remain unchanged.
2. Prediction: the probabilities of symptomatic remission for each patient are predicted by the trained classifier, both for the actual sample and the 48 counterfactual samples.
3. Computing the feature importance maps: for a given patient the feature importance map is compiled by computing the effect size for each of the 48 items. The effect size evaluates how having a certain comorbidity affects the probability of symptomatic remission in an individual patient. Therefore, the effect size for the *i*th item *C_i_* is computed by subtracting the predicted probability of remission in the actual/counterfactual scenario in which *C_i_ = “no”* from the predicted probability of remission in the counterfactual/actual scenario in which *C_i_ = “yes”* [i.e., *prob(remission|C_i_ = yes) – prob(remission|C_i_ = no)*].

The process of computing the feature importance maps is repeated for all patients in the test set and across different folds in 10-fold cross-validation. The result is a feature importance map for each patient, indicating the effect of each comorbidity item on the probability of remission. After computing these maps for all patients, we can (i) calculate the group-level feature importance (see section 3.2); and (ii) find sub-groups of patients with similar comorbidity effects on the symptomatic remission (see section 3.3). The statistical significance of the comorbidity effects at the group level was determined through Bonferroni-corrected Wilcoxon rank-sum tests using a bootstrapped null distribution.

## 3. Results

### 3.1. Including comorbidities: same prediction performance, but a better balance between sensitivity and specificity

**Figure 4:**
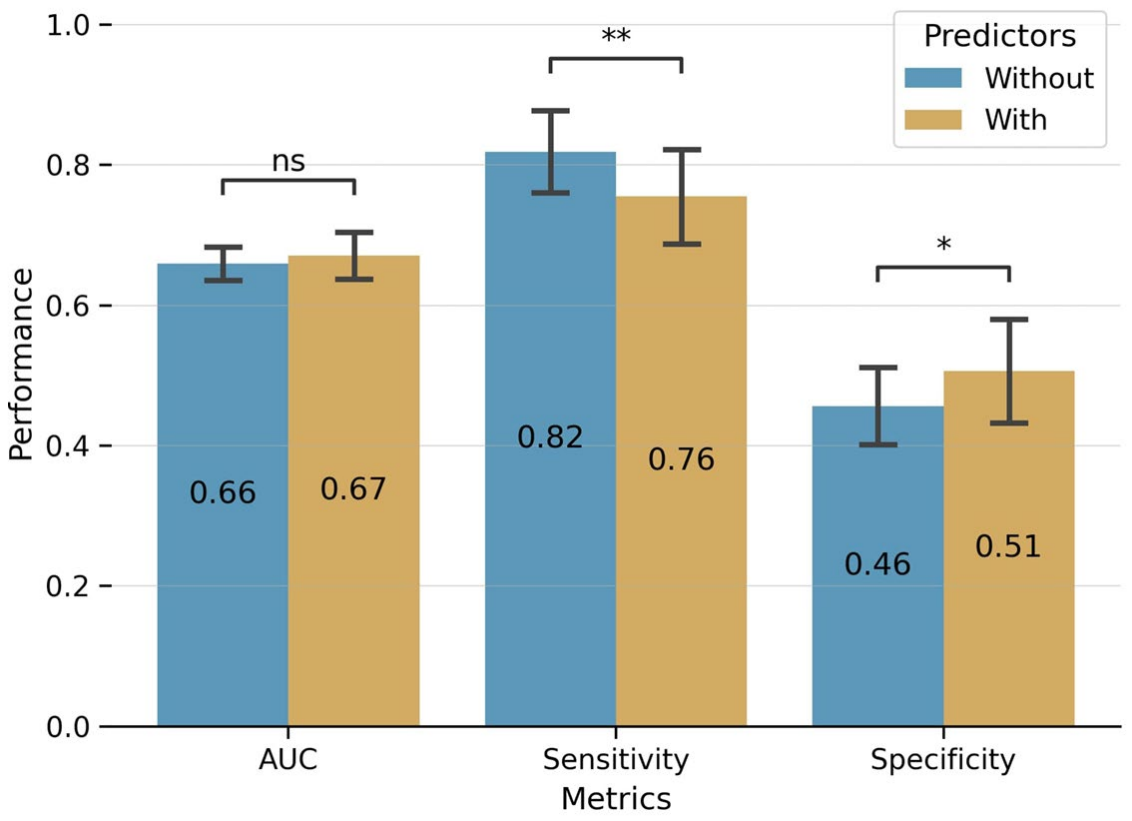
Comparison between the prediction performance of predictors without (blue bars) and with (orange bars) comorbidity features across 20 repetitions of 10-fold cross-validation. The predictor with comorbidity features shows a better balance between sensitivity and specificity. While there is no significant difference between the AUCs of the two predictors (0.66 ± 0.02 for the predictor without and 0.67 ± 0.03 for the predictor with comorbidity features), the predictor with comorbidity features shows significantly higher specificity compensating 0.06 of model sensitivity (ns: not significant, *: Mann–Whitney test *p*-value <0.05, **: Mann–Whitney test *p*-value <0.01).

Figure 4 depicts the comparative prediction performance of psychosis prognosis prediction models, both with and without MINI-PLUS comorbidity items, across 20 repetitions of 10-fold cross-validation. We employed the Mann–Whitney U test (24) to evaluate the statistical significance of the difference between the performance of the two models. The area under the curve (AUC) values for the two predictors do not exhibit a statistically significant difference (0.66 ± 0.02 for the predictor without comorbidity features and 0.67 ± 0.03 for the predictor with comorbidity features). However, the model with comorbidity features demonstrates a significantly (*p*-value<0.05) higher specificity, compensating for a 0.06 reduction in model sensitivity. The marginal improvement achieved by including comorbidity features in the prediction models may be attributed to their correlation with other included features. Nonetheless, the predictor with comorbidity features exhibits a more desirable balance between sensitivity and specificity, thereby enhancing its potential as a diagnostic clinical tool.

### 3.2. Negative associations between comorbidities and remission at group level

**Figure 5:**
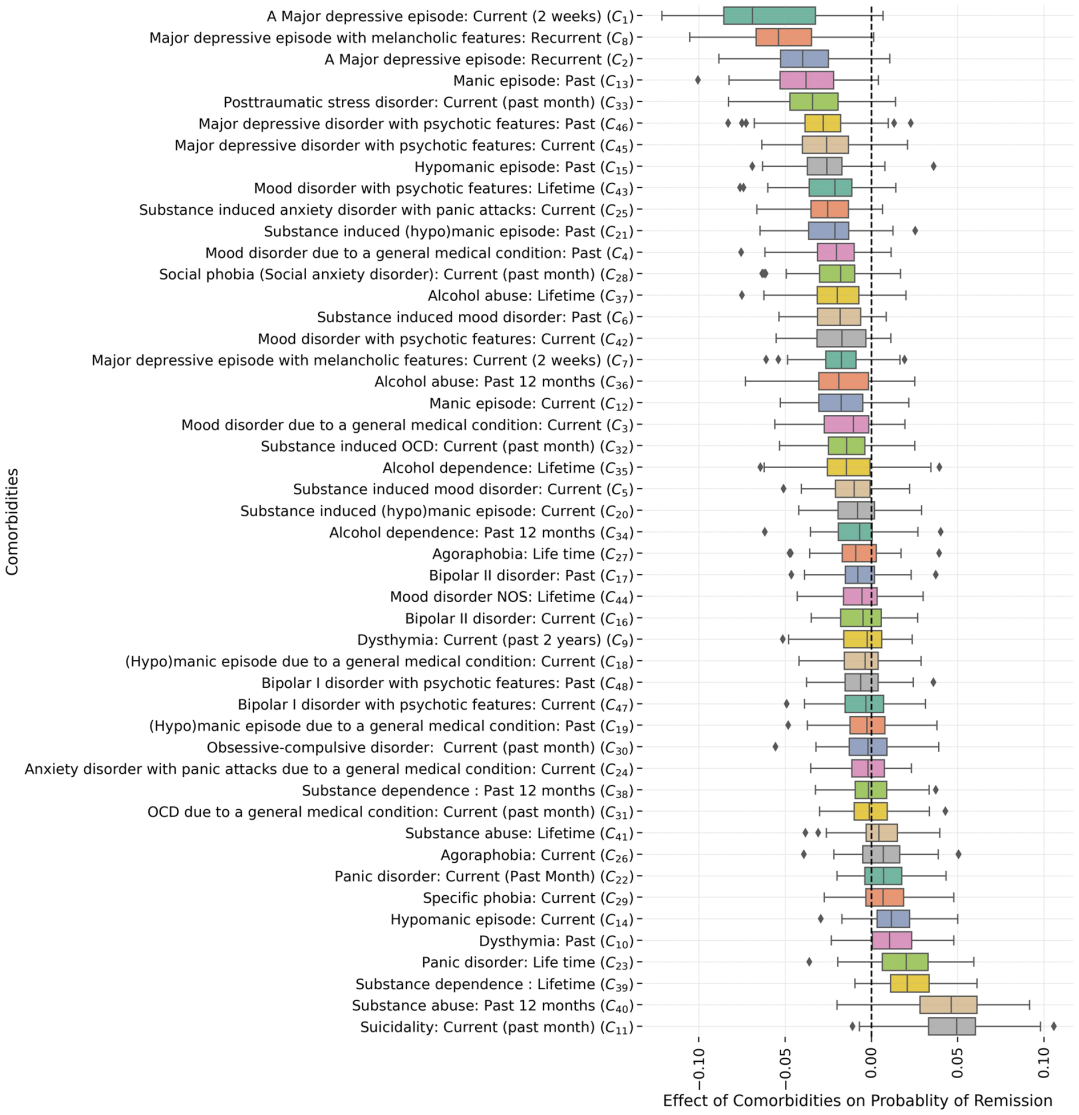
The sorted group effect of comorbidities on the probability of remission (x-axis) for 48 MINI binary comorbidity measures (y-axis). The boxplots present the median and quartiles for each item in the feature importance maps across patients. “Major depressive episode: current” (*C_1_*), “major depressive episode: recurrent” (*C_2_*), and “major depressive episode with melancholic features: recurrent” (*C_8_*) show a larger negative group effect with a median lower or close to −0.05 (i.e., the effect size is lower than −0.05 in at least half of patients). “Suicidality: current” (*C_11_*) and “substance abuse: past 12 months” (*C_40_*) show larger positive effects at the group level with a median higher or close to 0.05. All these 5 group effects are significantly different from the bootstrapped null distribution (Bonferroni corrected Wilcoxon rank-sum test *p*-value <0.001).

Figure 5 provides a comprehensive summary of the impact of 48 binary MINI comorbidity items on the likelihood of achieving remission at the group level. To facilitate interpretation, the items are arranged according to their effect in descending order from the most negative to the most positive. The results obtained at the group level reveal statistically significant negative group effects (*p*-value<0.001, Bonferroni-corrected) for “major depressive episode: current” with an average Wilcoxon test *r*-value of 0.66 (an *r*-value above 0.5 represents a large effect size), “major depressive episode: recurrent” with an average *r*-value of 0.64, “major depressive episode with melancholic features: recurrent” with an average *r*-value of 0.50. These effects, characterized by medians close to or below −0.05, demonstrate a pronounced influence on remission probability (scale: 0–1). Conversely, a few items, such as “suicidality: current” with an average *r*-value of 0.72 and “substance abuse: past 12 months” with an average *r*-value of 0.73, exhibit intriguingly positive effects on remission probability at the group level, with medians close to or above 0.05.

### 3.3. Comorbidity effects are heterogeneous but clustered at the individual level

**Figure 6:**
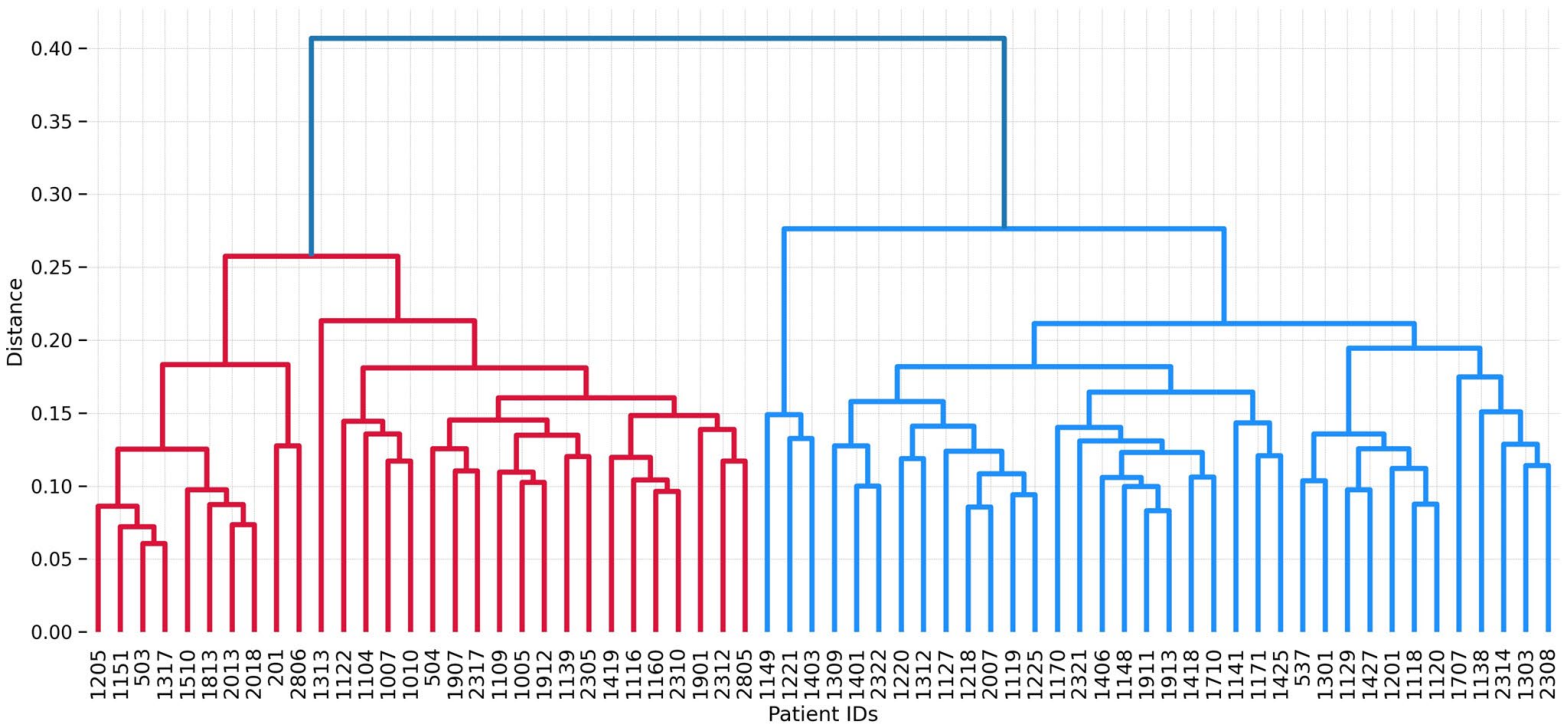
Hierarchical cluster analysis on the derived feature importance maps across patients (x-axis) reveals two major clusters. The probabilities of remission of 30 patients in the first cluster (red) are less affected by the comorbidities compared to 36 patients in the second (blue) cluster.

The analysis presented in Figure 5 reveals substantial variations in the impact of comorbidity items on the probability of achieving symptomatic remission among individual patients. To delve deeper into these variabilities, we employed a hierarchical clustering approach (25) on the feature importance maps derived from patients. For this purpose, we utilized the agglomerative clustering method with the ‘ward’ criterion for the linkage function, which aims to minimize the variance of the merged clusters. The outcomes of the hierarchical clustering are summarized in Figure 6, which indicates the presence of two prominent subgroups of patients exhibiting distinct profiles of comorbidity feature importance.

**Figure 7:**
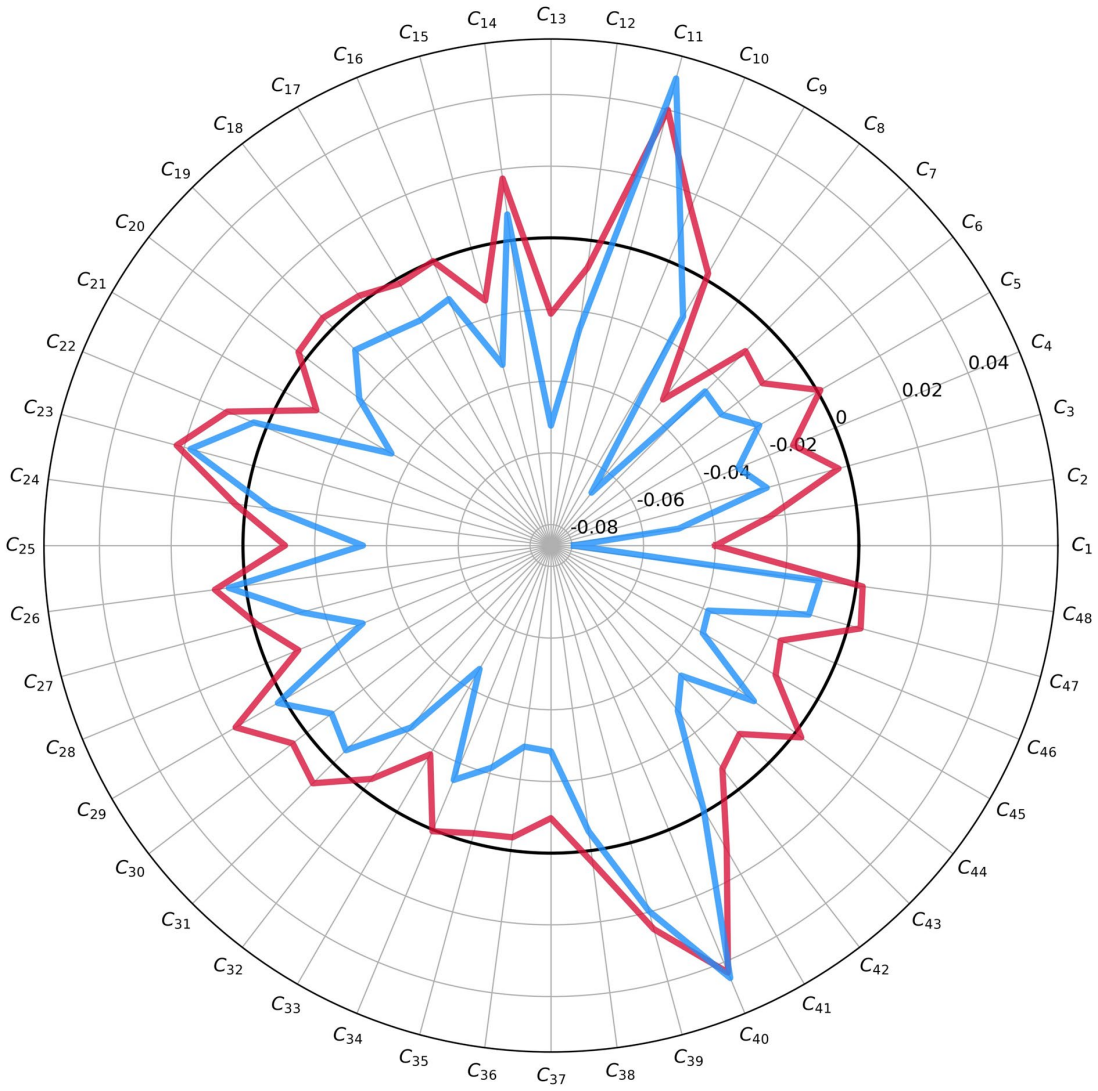
A comparison between average feature importance maps in two clusters in Figure 5. The average feature importance map of the red cluster is closer to zero compared to the blue cluster. This shows the smaller effect of comorbidity measures on the patients in the red clusters compared to the patients in the blue cluster.

To gain further insights into the similarities and dissimilarities within these two clusters, we computed the mean feature importance maps for each cluster. Figure 7 visualizes the results, highlighting the relatively diminished negative effect of comorbidity items on the predicted probability of remission in the red cluster, which encompasses approximately 45% of the patients. This contrast becomes evident when comparing these patients to those in the blue cluster.

## 4. Discussion

We investigated the impact of psychiatric comorbidity on outcomes in patients with early psychosis. Our analysis at the group level confirmed the previously established notion that most comorbidities have a negative influence on the predicted likelihood of symptomatic remission (3, 26). Notably, current and recurrent depressive disorder consistently exhibited the most pronounced negative impact on remission probability across patients. It is worth mentioning that while comorbid depressive symptoms and depressive disorder are widely recognized as significant negative prognostic factors of SSD, as they diminish the chances of functional remission (14) and lower quality of life (10, 15), our recent systematic review and meta-analysis did not find an association between depressive symptoms and symptomatic remission (27).

Interestingly, we observed a positive effect of suicidality in the past month on the likelihood of remission in patients. However, this association may be confounded by the level of insight into illness. Better insight has been identified as a positive predictor of symptomatic remission (27), but it is also a risk factor for suicidality in patients with SSD (28, 29). Another explanation of the positive effect of suicidality in the past month on the likelihood of remission may be found in the treatment of these patients; possibly, these patients are more often admitted and/or receive a more intense treatment (e.g., co-medication, higher doses), leading to an increased chance of remission. Further research is needed to elucidate this interaction between suicidality and the chance of remission.

Another unexpected finding was that substance abuse in the past 12 months positively influenced the chance of symptomatic remission in patients. Existing literature has provided inconclusive results regarding the association between comorbid substance abuse and symptomatic remission. A cross-sectional multicenter study involving 1,010 patients reported a lower likelihood of achieving symptomatic remission among substance abusers compared to non-abusers (OR 0.67) (30). However, a study based on routinely collected medical data from 608 patients revealed that individuals with SSD and comorbid substance abuse seemed to stabilize more rapidly during acute hospitalization compared to those without (31), which aligns with our findings. This observation might be explained by differences in the correlation between substance use and psychosis. Both clinically and in research it is difficult to differentiate between substance-induced psychosis and primary psychotic disorders with co-occurring substance abuse (32). Differentiation is further complicated by the fact that besides positive symptoms, drug effects can also mimic negative and cognitive symptoms (33). If psychotic symptoms are induced or exacerbated by substance use, they may dissipate rapidly upon discontinuation of the substance (31). However, the transition of a diagnosis of substance-induced psychosis to a primary psychotic disorder is common and occurs in about 25% of patients (34).

The findings at the individual level revealed significant variability among patients. Approximately 45% of the patients belonged to a subgroup where comorbidities had a relatively limited impact on the likelihood of remission. In contrast, the other subgroup exhibited a stronger influence of comorbidities on remission outcomes. The identification of two distinct groups of patients with differential effects of comorbidities on remission outcomes suggests the presence of underlying clinical heterogeneity within the patient population. This heterogeneity could stem from various factors such as differences in the severity or type of comorbidities, variations in treatment response, or diverse genetic and neurobiological factors.

Clinically, the variability of the impact of comorbidities on the likelihood of remission between patients has important implications. First, it highlights the need for personalized approaches to treatment and care. By recognizing the existence of subgroups with differing responses to comorbidities, clinicians can tailor interventions based on individual patient characteristics. This may involve adjusting medication regimens, implementing targeted psychotherapy, or addressing specific comorbid conditions to optimize remission outcomes.

Furthermore, identifying these distinct subgroups enables healthcare providers to better allocate resources and prioritize interventions. Patients in the subgroup where comorbidities have a limited effect on remission may require more focused attention on other factors influencing their outcomes, such as medication adherence, social support, or environmental factors. On the other hand, patients in the subgroup where comorbidities strongly impact remission may benefit from comprehensive management strategies that address both the primary condition and the comorbidities concurrently.

Overall, our study sheds light on the complex relationships between psychiatric comorbidity and outcomes in early psychosis. These findings underscore the importance of considering comorbidities when predicting and managing outcomes for patients with SSD. Further investigation is warranted to unravel the underlying mechanisms and inform the development of targeted interventions for patients with specific comorbidities.

### 4.1. Strengths and limitations

To our best knowledge, this is the first study that investigated psychiatric comorbidity as a predictor in a computerized prognostic prediction model for SSD.

One limitation of our study is the small number of patients. Our findings about the association between suicidality and substance abuse with remission are based on 14 patients (21% of the total sample) with substance abuse and 13 patients (20% of the total sample) with current suicidality. These small numbers increase the risk of chance findings. A second limitation is that we were not able to analyze the impact of somatic comorbidity as a predictor of remission alongside psychiatric comorbidity. Somatic comorbidity is common in patients with psychotic disorders, but little is known about a possible association between somatic comorbidities and the severity of psychotic symptoms and the efficacy of treatment. Previous research showed that somatic comorbidities were associated with better insight and more symptomatic improvement in a first-episode schizophrenia sample (34), while another study found that chronic somatic comorbidities were associated with higher rates of rehospitalization in patients with SSD (35). Unfortunately, the OPTiMiSE dataset did not include comprehensive information on somatic comorbidity. We expect a low incidence of somatic comorbidity in our study sample, based on the information gathered from the medication files and the fact that the study sample primarily comprised young individuals with first-episode psychosis. This demographic is generally associated with better physical health due to their age. Due to the lack of data on somatic comorbidity, we could not determine whether somatic comorbidity is a confounder in the investigated association between psychiatric comorbidity in SSD and the chance of symptomatic remission in our sample. A third limitation is that the effect of comorbidity was tested independently without considering potential interactions with other features such as age, lifestyle factors, family history, and symptoms of SSD including insight into illness. Further research could explore the interplay between comorbidity and other factors to gain a more comprehensive understanding of their combined impact on prognosis.

### 4.2. Meaning of the results

Our findings highlight the significance of considering psychiatric comorbidities when predicting prognosis in schizophrenia spectrum disorders (SSD). However, it is essential to acknowledge that our study does not provide insight into the underlying nature of the association between SSD and psychiatric comorbidities.

There are several potential explanations for the impact of psychiatric comorbidities on the prognosis of SSD. Firstly, the correlation between SSD and psychiatric comorbidity may be attributed to a shared genetic vulnerability for psychiatric disorders. This heightened genetic susceptibility could exert a negative influence on prognosis. Secondly, psychiatric comorbidities might arise as reactions to SSD, indicating a more severe disease and contributing to unfavorable outcomes. For example, depression could emerge as a reaction to the diagnosis of schizophrenia. Thirdly, psychiatric comorbidities could manifest as side effects of SSD treatment, thereby limiting treatment options and potentially exacerbating prognosis. An example of this is the development of obsessive-compulsive disorder as a side effect of clozapine use, which may necessitate discontinuation of the medication and subsequently worsen the prognosis of SSD. Lastly, if the comorbid psychiatric disorder is viewed as an independent condition, the increased burden of disease may have a detrimental impact on prognosis. In such cases, treating psychiatric comorbidity could potentially improve the prognosis of SSD. However, we did not find studies investigating the effect of treating psychiatric comorbidity on the prognosis of SSD.

Further research is necessary to delve into these potential explanations and to explore the impact of treating psychiatric comorbidity on the prognosis of SSD.

## 5. Conclusion

By using complex machine learning models and a counterfactual model explanation technique, we showed that at the group level, most psychiatric comorbidities have a negative influence on the predicted likelihood of symptomatic remission in schizophrenia spectrum disorders. At the individual level, we found high variability in the influence of the presence of comorbidities on the chance of remission. These findings highlight the importance of identifying and including relevant comorbidities in prediction models and provide valuable insights for improving the treatment and prognosis of individual patients with psychotic disorders.

## Data availability statement

The data analyzed in this study is subject to the following licenses/restrictions: there is no consent to make the data publicly available. Requests to access these datasets should be directed to IW-vR, i.winter@umcutrecht.nl.

## Ethics statement

Ethical approval was not required for the study involving humans in accordance with the local legislation and institutional requirements. Written informed consent to participate in this study was not required from the participants or the participants’ legal guardians/next of kin in accordance with the national legislation and the institutional requirements.

## Author contributions

SK, VD, HS, and WC contributed to the conception and design of the study and drafted the manuscript. RK and IW-vR provided the data and organized the database. SK carried out the modeling and statistical analysis. All authors contributed to the article and approved the submitted version.
