# Supplementary material for: Revealing the impact of psychiatric comorbidities on treatment outcome in early psychosis using counterfactual model explanation
Source: Front Psychiatry. 2023 Oct 12;14:1237490. doi: 10.3389/fpsyt.2023.1237490 (PMC10602778; doi:10.3389/fpsyt.2023.1237490)
Supplement: Supplementary file 1 [file Table_1.pdf]

Supplementary Table S1

Binary MINI comorbidity measures used in this study (ID: the identifier for each comorbidity feature in our experiments, Field Name: the field name in OPTiMiSE dataset).

ID	Field Name	Description
C_1	V1_MINI_A_DEPR_CURR	A Major depressive episode: Current (2 weeks)
C_2	V1_MINI_A_DEPR_RECURR	A Major depressive episode: Recurrent
C_3	V1_MINI_A_DPMOOD_GNR_CURR	Mood disorder due to a general medical condition: Current
C_4	V1_MINI_A_DPMOOD_GNR_PAST	Mood disorder due to a general medical condition: Past
C_5	V1_MINI_A_DPMOOD_SUBST_CURR	Substance induced mood disorder: Current
C_6	V1_MINI_A_DPMOOD_SUBST_PAST	Substance induced mood disorder: Past
C_7	V1_MINI_A_MELANCH_CURR	Major depressive episode with melancholic features: Current (2 weeks)
C_8	V1_MINI_A_MELANCH_RECURR	Major depressive episode with melancholic features: Recurrent
C_9	V1_MINI_B_DYST_CURR	Dysthymia: Current (past 2 years)
C_{10}	V1_MINI_B_DYST_PAST	Dysthymia: Past
C_{11}	V1_MINI_C_CURR	Suicidality: Current (past month)
C_{12}	V1_MINI_D_MANIC_CURR	Manic episode: Current
C_{13}	V1_MINI_D_MANIC_PAST	Manic episode: Past
C_{14}	V1_MINI_D_HYPOM_CURR	Hypomanic episode: Current
C_{15}	V1_MINI_D_HYPOM_PAST	Hypomanic episode: Past
C_{16}	V1_MINI_D_BIPOLII_CURR	Bipolar II disorder: Current
C_{17}	V1_MINI_D_BIPOLII_PAST	Bipolar II disorder: Past
C_{18}	V1_MINI_D_MANIC_GNR_CURR	(Hypo)manic episode due to a general medical condition: Current
C_{19}	V1_MINI_D_MANIC_GNR_PAST	(Hypo)manic episode due to a general medical condition: Past
C_{20}	V1_MINI_D_MANIC_SUBST_CURR	Substance induced (hypo)manic episode: Current
C_{21}	V1_MINI_D_MANIC_SUBST_PAST	Substance induced (hypo)manic episode: Past
C_{22}	V1_MINI_E_PANIC_CURR	Panic disorder: Current (Past Month)
C_{23}	V1_MINI_E_PANIC_LIFE	Panic disorder: Life time

C_{24}	V1_MINI_E_PANIC_GNR_CURR	Anxiety disorder with panic attacks due to a general medical condition: Current
C_{25}	V1_MINI_E_PANIC_SUBST_CURR	Substance induced anxiety disorder with panic attacks: Current
C_{26}	V1_MINI_F_AGORA_CURR	Agoraphobia: Current
C_{27}	V1_MINI_F_AGORA_LIFE	Agoraphobia: Life time
C_{28}	V1_MINI_G_SOC_CURR	Social phobia (Social anxiety disorder): Current (past month)
C_{29}	V1_MINI_H_SPEC_CURR	Specific phobia: Current
C_{30}	V1_MINI_I_OBSCOMP_CURR	Obsessive-compulsive disorder: Current (past month)
C_{31}	V1_MINI_I_OCD_GNR_CURR	OCD due to a general medical condition: Current (past month)
C_{32}	V1_MINI_I_OCD_SUBST_CURR	Substance induced OCD: Current (past month)
C_{33}	V1_MINI_J_PTSTRESS_CURR	Posttraumatic stress disorder: Current (past month)
C_{34}	V1_MINI_K_ALCDEP_12M	Alcohol dependence: Past 12 months
C_{35}	V1_MINI_K_ALCDEP_LIFE	Alcohol dependence: Lifetime
C_{36}	V1_MINI_K_ALCABS_12M	Alcohol abuse: Past 12 months
C_{37}	V1_MINI_K_ALCABS_LIFE	Alcohol abuse: Lifetime
C_{38}	V1_MINI_L_SUBSTDEP_12M	Substance dependence : Past 12 months
C_{39}	V1_MINI_L_SUBSTDEP_LIFE	Substance dependence : Lifetime
C_{40}	V1_MINI_L_SUBSTABS_12M	Substance abuse: Past 12 months
C_{41}	V1_MINI_L_SUBSTABS_LIFE	Substance abuse: Lifetime
C_{42}	V1_MINI_M_MOOD_CURR	Mood disorder with psychotic features: Current
C_{43}	V1_MINI_M_MOOD_LIFE	Mood disorder with psychotic features: Lifetime
C_{44}	V1_MINI_M_MOODNOS_LIFE	Mood disorder NOS: Lifetime
C_{45}	V1_MINI_M_MAJDEP_CURR	Major depressive disorder with psychotic features: Current
C_{46}	V1_MINI_M_MAJDEP_PAST	Major depressive disorder with psychotic features: Past
C_{47}	V1_MINI_M_BIPOLI_CURR	Bipolar I disorder with psychotic features: Current
C_{48}	V1_MINI_M_BIPOLI_PAST	Bipolar I disorder with psychotic features: Past
